# Investigating the Influence of Drying Methods on the Quality and Bioactive Properties of Hemp ( *Cannabis sativa* L.) Seed Oil

**DOI:** 10.1002/fsn3.70894

**Published:** 2025-09-01

**Authors:** Oumayma Boussetta, Amal Elrherabi, Fahd A. Nasr, El Hassania Loukili, Meryem Idrissi Yahyaoui, Nouhaila Amrani, Mohamed Chebaibi, Abdeslam Asehraou, Mohamed Bouhrim, Mohammed Al‐zharani, Ashraf Ahmed Qurtam, Mohammed Ramdani

**Affiliations:** ^1^ Laboratory of Applied Chemistry and Environment, Faculty of Science University Mohammed 1st Oujda Morocco; ^2^ Laboratory of Bioresources, Biotechnology, Ethnopharmacology and Health, Faculty of Sciences University Mohammed 1st Oujda Morocco; ^3^ Biology Department, College of Science Imam Mohammad Ibn Saud Islamic University (IMSIU) Riyadh Saudi Arabia; ^4^ Euromed University of Fes, UEMF FES Morocco; ^5^ Ministry of Health and Social Protection Higher Institute of Nursing Professions and Health Techniques Fez Morocco; ^6^ Laboratory of Biological Engineering, Team of Functional and Pathological Biology; Faculty of Sciences and Techniques Beni Mellal University Sultan Moulay Slimane Beni Mellal Morocco

**Keywords:** antimicrobial activity, antioxidant activity, *Cannabis sativa* L. seeds, gas chromatography–mass spectrometry (GC/MS), microwave drying, oil yield, oven drying

## Abstract

This research investigates how oven and microwave drying methods influence mass reduction, oil yield, chemical composition, and bioactivity of *Cannabis sativa* seed oil, from two *C. sativa* L. varieties—Beldia and Critical—grown in Ketama and Taounate, Morocco. Microwave drying resulted in greater mass loss (2.1%–1.8%) compared to oven drying (1.48%–1.1%), but reached moisture stabilization within just 25 min, significantly faster than the 21 h needed for oven drying. Drying enhanced oil yield, with the highest recorded at 23.12% in oven‐dried Ketama Critical seeds and 23% in microwave‐dried Taounate Critical seeds, whereas untreated seeds yielded between 18.75% and 20.36%. GC–MS analysis identified linoleic acid as the predominant fatty acid, comprising 79.85% in fresh Ketama Beldia seeds and decreasing to 66.40% (oven‐dried) and 79.27% (microwave‐dried). Antioxidant capacity, assessed via the DPPH assay, peaked in the non‐dried Ketama Beldia oil (IC_50_ = 9.617 μg/mL), surpassing even ascorbic acid (IC_50_ = 61.34 μg/mL). However, drying significantly diminished antioxidant activity, with IC_50_ values rising to 31.185 μg/mL (oven), and 20.377 μg/mL (microwave). Antibacterial tests revealed inhibition zones ranging from 11 to 15.7 mm, with the microwave‐dried Taounate Beldia variety showing the strongest effect. Antifungal assays also indicated improved activity post‐drying, with a minimum inhibitory concentration (MIC) of 0.25% against *Geotrichum candidum* in oven‐dried Taounate Beldia seeds. These findings highlight the potential contribution of Cannabis sativa seed oil to food security through its nutritional and bioactive properties.

## Graphical abstract

This study investigates the effect of climate change on the chemical composition and biological activities of *Inula viscosa* essential oils. Essential oils from plants cultivated under simulated climate scenarios showed variation in yield and composition. The oils exhibited promising antioxidant, antidiabetic, and molecular docking‐based bioactivity, especially under moderate climate stress, revealing their potential as therapeutic and eco‐friendly agents.

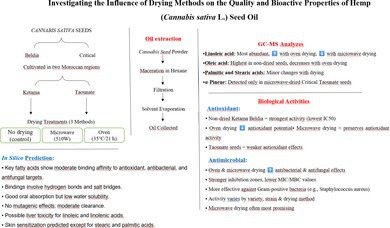

## 1 Introduction

*Cannabis sativa* L. (Hemp) is an annual herb of the Cannabaceae family that is considered native to Central Asia (Pieracci et al. 2021). Hemp cultivation is one of humanity's earliest cultures. It has been widely farmed and utilized throughout history for its nutritional and medicinal properties before being used for its psychoactive effect caused by the Δ9‐tetrahydrocannabinol (Δ9‐THC) molecule (Chen et al. 2010), which is distributed differently in the plant parts. It is abundant in female flowers, present at low concentrations in leaves and stems, and completely absent from seeds and roots (Glivar et al. 2020).

*Cannabis sativa* L. contains diverse chemical compounds, with current research identifying over 500 substances within the plant. Among these, more than 140 are classified as cannabinoids, the primary active constituents responsible for many plant effects. In addition to cannabinoids, *C. sativa* L. encompasses a variety of other chemical classes, including terpenes; approximately 120 compounds contributing to the plant's aroma and potential therapeutic properties, phenolic and polyphenolic compounds; around 46 compounds known for their antioxidant activities, sugars; about 34 different types present in the plant, and ketones and aldehydes; approximately 25 compounds identified (Jin et al. 2020). Hemp seeds are characterized by their nutritional chemical composition, rich in polyunsaturated, monounsaturated, and saturated fatty acids, carbohydrates, proteins, mineral elements, and many bioactive compounds (Farinon et al. 2020). *C. sativa* L. seed oil presents an omega‐6/omega‐3 ratio between 3:1 and 5:1, ideal for reducing cardiovascular disease risk (Simopoulos 2008; Haddou et al. 2024). Biological activities associated with *C. sativa* L. oil are diverse and have been the focus of numerous studies. The presence of cannabinoids, particularly CBD, has been linked to various health benefits, including anti‐inflammatory, analgesic, anticonvulsant, antipyretic, anxiolytic, and antibacterial activities (Chouvy 2008; Haddou, Hassania Loukili, et al. 2023). Research suggests that these compounds may interact with the endocannabinoid system in the human body, potentially aiding in the management of conditions such as chronic pain, anxiety disorders, and epilepsy. Furthermore, the oil's rich fatty acid profile supports cardiovascular health and skin wellness, making it a valuable addition to dietary and cosmetic products (Frassinetti et al. 2018).

The thermal treatment of the seeds is a key process that can influence the texture, color, flavor, and overall appearance of seeds, as it enhances both the yield and the nutritional quality of the oil (Chandrasekara and Shahidi 2011; Hama 2017). Previous studies have demonstrated how drying techniques, such as hot air or microwave drying can significantly affect the phytochemical composition, physical attributes, and biological activities of various plant matrices, including banana slices and citrus peels. These findings provide a rationale for exploring the optimization of drying parameters to preserve or enhance the functional properties of plant‐based oils. Moreover, drying modes cause significant physical, chemical, and sensory changes in seeds. Although roasting can improve phenolic release by breaking covalent bonds between phenolic compounds and the cell wall, it also accelerates the degradation of seed stability compared to fresh seeds (Dewanto et al. 2002). Drying treatments also alter the phenolic profile, potentially boosting their antioxidant capacity and health benefits (Kim et al. 2011).

In this study, we conducted a comparative investigation into the effects of oven and microwave drying methods on the mass loss, oil yield, chemical composition, and biological activities of hexane‐extracted oils from two distinct varieties of *C. sativa* L. seeds: Beldia (B) and Critical (C). These varieties were harvested from two ecologically diverse regions in Morocco, Ketama (K) and Taounate (T). By linking the drying method, seed variety, and regional origin, this study provides a novel framework for optimizing the extraction of bioactive compounds and enhancing specific biological activities. Furthermore, the research introduces a systematic approach to tailoring drying parameters to maximize desirable chemical profiles and bioactivity outcomes.

Therefore, this study aims to evaluate how different drying methods (oven and microwave) influence the chemical composition, oil yield, and biological activities (antioxidant and antimicrobial) of hemp seed oil extracted from two *C. sativa* L. varieties (Beldia and Critical) grown in distinct Moroccan regions. We hypothesize that both the drying method and the varietal origin significantly impact the phytochemical content and bioactivity of the resulting oils, with potential implications for nutrition improvement and the valorization of hemp seed oil as a functional food ingredient.

## 2 Materials and Methods

### 2.1 Plant Material

In this study, we used two varieties of *C. sativa* L. seeds: Beldia (B) and Critical (C) cultivated in September 2022 in two distinct regions of Morocco: Ketama (K) (Latitude: 34.9158, Longitude: −4.5685634°54′57″ North, 4°34′7″ West. Altitude: 1505 m), and Taounate (T) (Latitude: 34.5358, Longitude: −4.6434°32′9″ Nord, 4°38′24″ Ouest. Altitude: 592 m). The Beldia and Critical varieties were chosen due to their distinct characteristics and local significance. Beldia is a traditional landrace valued for its richness in bioactive compounds, whereas Critical is known for its agronomic performance and chemical potential. The Ketama and Taounate regions were selected for their favorable climates and long‐standing reputations in *Cannabis* cultivation, ensuring representative and diverse samples for the study.

Prior to drying, all harvested seeds were visually inspected to ensure uniform maturity based on color and firmness. Only fully mature seeds were selected for analysis. To minimize variability, seeds were sieved to obtain a uniform size distribution and equilibrated at room temperature for 24 h to standardize initial moisture content before the drying treatments.

To investigate the effect of the drying mode on the chemical composition of the seed's extracts, we divided our samples into three categories. The first category consisted of seeds used directly after cultivation without drying, serving as the control. In the second category, seeds were dried using microwaves (510 W) for 21 to 35 min with 1‐min intermittent cooling, continuing until weight loss stabilized. For the third category, seeds were dried in an oven at 35°C for 21 h. The resulting 12 seed samples were then ground into a powder.

### 2.2 Oil Extraction

*Cannabis sativa* L. oil was extracted using the maceration technique. Specifically, 20 g of seed powder was mixed with 60 mL of hexane and stirred magnetically for 3 h at room temperature (25°C). The mixture was then filtered three times using No. 1 filter paper. The resulting filtrate was processed with a rotary evaporator to remove the solvent and recover the seed oil, which was stored at 4°C until use.

### 2.3 Phytochemical Study

The separation and identification of fatty acid methyl esters (FAMEs) were performed using a Shimadzu GC system (Kyoto, Japan) equipped with a BPX25 capillary column containing 5% of diphenyl and 95% of dimethylpolysiloxane phase (30 m × 0.25 mm inner diameter × 0.25 μm film thickness), coupled to a QP2010 MS. High‐purity helium gas (99.99%) was used as carrier gas with a constant flow rate of 3 mL/min. The injection, ion source, and interface temperatures were all set at 250°C. The temperature program for the column oven was 50°C (held for 1 min), heated to 250°C at 10°C/min, and held for 1 min. The ionization of the sample components was done in the EI mode (70 eV). The mass range scanned was 40–300 *m*/*z*. One microliter of each prepared extract diluted with an appropriate solvent was injected in a splitless mode (split ratio 90: 1). All samples were analyzed in triplicate. Finally, compounds were identified by comparison of their retention times with those of authentic standards and their mass spectrum fragmentation patterns with those found in databases or those stored on the National Institute of Standards and Technology (NIST) 147, 198 compounds. LabSolutions (version 2.5) was used for data collection and processing (Kadda et al. 2022; Taibi et al. 2024; Loukili et al. 2021).

### 2.4 Biological Activities

#### 2.4.1 DPPH Test

The antioxidant activity of *C. sativa* L. seed oil is tested using the DPPH (2,2‐Diphenyl‐1‐Picrylhydrazyl) method. The free radical neutralizing ability of hexane extracts from cannabis seeds was tested in triplicate. In brief, 0.2 mL of each extract, at varying concentrations (0.032, 0.062, 0.125, 0.250, 0.500, 1, and 2 mg/mL) dissolved in methanol, was mixed with 1.8 mL of a methanolic DPPH solution (4 mg of DPPH in 100 mL of methanol). The mixture was vortexed for 60 s and then incubated in the dark at room temperature for 20 min. Absorbance was measured at 517 nm using an Ultrospec 7000 UV–visible spectrophotometer. Under similar conditions, ascorbic acid was the reference standard (positive control) (Laaroussi, Aouniti, et al. 2022; Miri and Djenane 2019).

The DPPH radical neutralizing potential of the extracts was then determined based on the following formula:

$$\text{Inhibition}\;\left(\%\right)=\left(\left(A_{\text{control}}-A_{\text{test}}\right)/A_{\text{control}}\right)\times 100$$

The level of DPPH inhibition by the extracts was expressed as the percentage of concentration required to achieve 50% inhibition (IC_50_) (El Guerrouj et al. 2023; Laaroussi et al. 2022).

#### 2.4.2 Total Antioxidant Capacity (TAC) Test

The total antioxidant capacity of hemp seed hexane extracts was evaluated using the phosphomolybdenum method. This technique is based on the reduction of molybdenum (VI) ions (MoO4^2−^) to molybdenum (V) (${\mathrm{MoO}}_{2}^{+}$) in the presence of the extract, forming a green phosphate/Mo (V) complex under acidic conditions (Pellegrini et al. 2021).

A volume of 0.3 mL of each concentration (0.032, 0.062, 0.125, 0.250, 0.500, 1, and 2 mg/mL) of methanolic samples was mixed with 0.6 mL of the reagent solution (0.6 M sulfuric acid, 28 mM sodium phosphate, and 4 mM ammonium molybdate). The tubes and the blank were incubated at 95°C for 90 min. After cooling, the absorbance of the solutions was measured at 695 nm against the blank. The TAC is expressed in milligrams of ascorbic acid equivalent per gram of dry matter (mg AAE/g DM). The test was performed in triplicate.

Results are expressed in terms of EC_50_, which refers to the extract concentration required to achieve 50% inhibition of oxidative activity.

#### 2.4.3 Antibacterial Activity

Before testing, the extracted oil was diluted in 2% dimethyl sulfoxide (DMSO) to ensure better dispersion in aqueous media. The oil‐DMSO solution was then sterilized by passing it through a 0.22 μm syringe filter to avoid introducing microbial contaminants during the assays. We examined antibacterial efficacy against two Gram‐positive strains— *Staphylococcus aureus* ATCC 6538P and *Micrococcus luteus* LB 14110—and two Gram‐negative strains— *Pseudomonas aeruginosa* ATCC 15442 and *Escherichia coli* ATCC 10536. Antifungal effectiveness was measured against *Candida glabrata*, *Rhodotorula glutinis* ON 209167, *Aspergillus niger*, and *Geotrichum candidum*. We tested concentrations from 16% to 0.125% to find the minimum inhibitory concentration (MIC). The method involved 96‐well microplates and the broth microdilution process, as described by Balouiri et al. (2016). We added 50 μL of the microbial inocula standardized to 10^5^ cells/mL. Positive controls matched the microorganisms—tetracycline for bacteria and cycloheximide for fungi. The plates incubated at 37°C for 24 h for bacteria and at 25°C for 48 h for fungi were later augmented with 15 μL of 0.015% resazurin and incubated further for 2 h to check metabolic activity. A change from blue resazurin to pink resorufin indicated viability (Lekbach et al. 2018). Each test was conducted in triplicate for precision.

#### 2.4.4 Antifungal Activity

Four fungal strains were selected to assess the antifungal activity of *C. sativa* L.: *R. glutinis*, *A. niger*, *C. glabrata*, and *G. candidum*. These strains were obtained from the Laboratory of Bioresources, Biotechnology, Ethnopharmacology, and Health at the Faculty of Science, Oujda, Morocco. The fungal cultures were maintained on Sabouraud Dextrose Agar at 28°C and regularly subcultured to ensure viability.

### 2.5 Molecular Docking

#### 2.5.1 Ligand Preparation

All compounds identified in the hexane extract of *C. sativa* L. Seed were downloaded in SDF format from the PubChem database. These ligands were subsequently processed and optimized using Schrödinger's LigPrep tool (version 11.5) under the OPLS3 force field. The optimization included adjustments for ionization states at a physiological pH range of 7.0 ± 2.0, allowing for the creation of up to 32 stereoisomeric variants per compound (Bouslamti et al. 2023; Chebaibi, Bourhia, et al. 2024).

#### 2.5.2 Protein Preparation

The Protein Data Bank was used to download the crystal structures of human NADPH oxidase (PDB ID: 2CDU), beta‐ketoacyl‐[acyl carrier protein] synthase from *E. coli* (PDB ID: 1FJ4), *S. aureus* nucleoside diphosphate kinase (PDB ID: 3Q8U), and *G. candidum* Cel7A structure (PDB ID: 4ZZT). These structures were prepared through a series of steps, including adding hydrogen atoms, correcting bond orders, removing water molecules, assigning hydrogen bonds, optimizing receptor atom charges, and energy minimization using the OPLS3 force field (Cherriet et al. 2023; Amrati et al. 2023; Tourabi et al. 2023).

#### 2.5.3 Glide Standard Precision (SP) Ligand Docking

Ligand docking was performed flexibly using the Standard Precision (SP) protocol within the Glide tool of Schrödinger‐Maestro (version 11.5). To ensure accurate modeling, non‐cis/trans amide bond configurations were penalized during the procedure. The Van der Waals forces for ligand atoms were adjusted with a scaling factor of 0.80, and a partial charge cutoff of 0.15 was applied. Docking results were analyzed using glide scores calculated from the energy‐optimized ligand conformations. The conformation with the most favorable (lowest) glide score was identified as the optimal binding pose for each ligand (Beniaich et al. 2022; Chebaibi, Mssillou, et al. 2024).

### 2.6 ADME/ Toxicity Analyses

The physicochemical characters and ADMET parameters of the molecules identified in the hexane extract of *C. sativa* L. Seed were carried out using online tools, such as http://www.swissadme.ch/ and https://biosig.lab.uq.edu.au/pkcsm/prediction.

The physicochemical parameters analyzed using the SWISSADME site include molecular weight (MW), number of rotatable bonds, hydrogen bond acceptors and donors, molar refraction (MR), topological polar surface area (TPSA), and WLOGP partition coefficient. The assessment of their similarity to drugs was performed using bioavailability radars, taking into account six key parameters for the design of oral drugs: lipophilicity, polarity, size, solubility, saturation, and flexibility (Ritchie et al. 2011; Daina et al. 2017).

Regarding the pharmacokinetics parameters, several parameters were examined regarding absorption, including water solubility, permeability through Caco‐2 cells, intestinal absorption in humans, skin permeability, and interaction with P‐glycoprotein, assessing whether the compounds are substrates or inhibitors of its isoforms I and II. Concerning distribution, we analyzed the apparent volume of distribution (VDss) in humans, the fraction not bound to plasma proteins, and the permeability to the blood–brain barrier (BBB) and the central nervous system (CNS). For metabolism, the study focused on identifying substrates and inhibitors of the main cytochrome P450 enzymes, including CYP2D6, CYP3A4, CYP1A2, CYP2C19, and CYP2C9. Excretion was assessed by analyzing total clearance and interaction with the renal transporter OCT2 (Lohohola et al. 2021).

Furthermore, toxicity represents a determining parameter in the drug design process. In our study, several toxicity criteria were considered, including mutagenicity assessed by the Ames test, maximum tolerated dose in humans, inhibition of hERG I and II channels, acute oral toxicity in rats (LD_50_), chronic oral toxicity in rats (LOAEL), hepatotoxicity, skin sensitization, as well as toxicity on *Tetrahymena pyriformis* and fish (Minnow toxicity) (Azzam 2023).

### 2.7 Statistical Analysis

All data are presented as means ± standard deviations. One‐way analysis of variance (ANOVA) was used to assess the significance of differences among groups. When significant differences were detected (*p* < 0.05), Tukey's Honest Significant Difference (HSD) post hoc test was applied to determine specific group differences. Statistical analyses were performed using STATISTICA software (version 7.1). A *p* value < 0.05 was considered statistically significant.

## 3 Results and Discussion

### 3.1 Mass Loss

**FIGURE 1:**
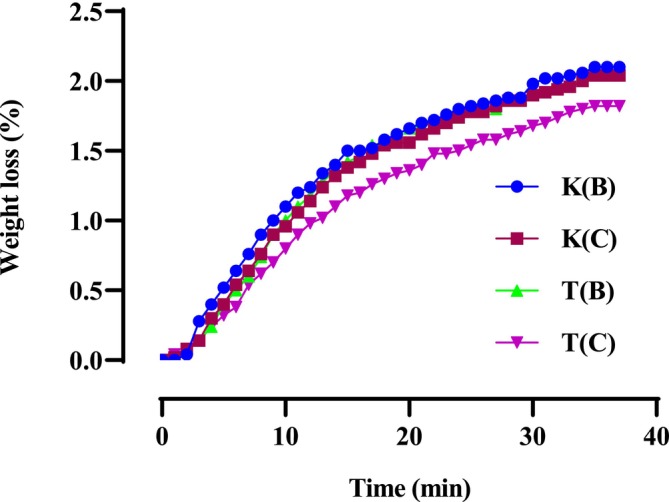
Mass loss of microwave‐dried seeds.

**FIGURE 2:**
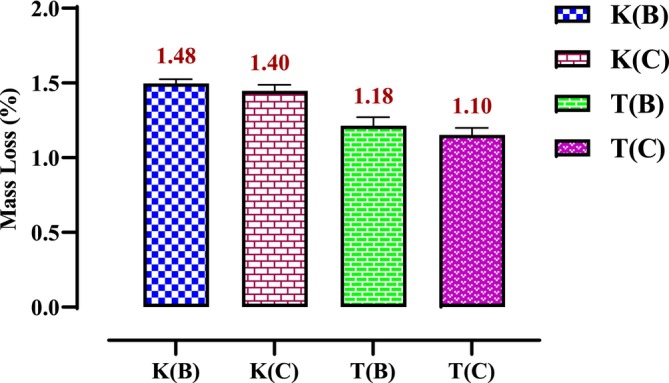
Mass loss of oven‐dried seed.

The mass loss of microwave‐dried seeds is significantly higher than that of oven‐dried seeds. Figure 1 indicates that the moisture content of microwave‐dried hemp seeds ranges from 2.1% to 1.8% for the Ketama Beldia (K(B)) and Taounate Beldia (T(B)) varieties, respectively. In comparison, the moisture content of oven‐dried seeds ranges from 1.48% to 1.1% for the Ketama Beldia (K(B)) and Taounate Critical (T(C)) varieties, as illustrated in Figure 2.

The results reveal that variety T(B), when dried in the microwave, experiences the lowest mass loss compared to other varieties. It stabilizes in a relatively short time (around 25 min). In contrast, the mass losses of other varieties do not stabilize until after 35 min.

The differences in mass loss and stabilization times between microwave‐dried and oven‐dried seeds arise primarily from the mechanisms of heat transfer and water removal in the two methods. Microwave drying is faster and suitable for time‐critical applications, but it requires careful control to prevent overheating and uneven moisture distribution. For this reason, we chose to work at 520 W with 1‐min intermittent cooling intervals to ensure the seed quality was not adversely affected. On the other hand, oven drying, although slower, provides more uniform drying and is better suited for preserving seed viability.

According to a study evaluated by B.D., Oomah et al., on the properties of hemp seed oil ( *C. sativa* L.), drying the seeds progressively increases weight loss. This increase follows a curve plotted against time, corresponding to a power law model when the data is regressed based on drying time (Oomah et al. 2002). Overall, microwave drying increases weight loss, aligning with the expected range for hemp seeds, as reported by Mölleken and Theimer (1997).

### 3.2 Extraction Yield

The extraction yield from non‐dried (N‐D) *C. sativa* seeds is notably high in the hexane extract, reaching approximately 20%. However, drying the seeds significantly increases the yield. For seeds cultivated in the Ketama region, oven (Ov) drying resulted in the highest yield, with a maximum value of 23.12% observed for the Critical K(C) variety. Conversely, for seeds from the Taounate region, microwave (Mw) drying proved to be more effective, leading to improved hexane extraction yields (Table 1).

**TABLE 1:** Hexane extract yields based on the seed drying method, in percentage (%).

	K(B)	K(C)	T(B)	T(C)
N‐D	18.75	20.12	19.63	20.36
Ov	21.90	23.12	21.42	20.64
Mw	20.489	22.36	22.56	23

By comparing these results with previous research on different extracts of *C. sativa* L. seeds from Morocco, it was found that the hexane extract in that study yielded 11.76%, which is relatively low compared to the yields observed in our study (Haddou, Hassania Loukili, et al. 2023). Another survey by Ismail et al. demonstrated that the extract from *C. sativa* L. seeds of the ‘Critical Plus’ cultivar showed a significant superiority in essential oil yield (El Bakali et al. 2022). Furthermore, Taoufik et al. (2017) conducted a study on the chemical characterization of hemp seed oils from three varieties cultivated in Morocco, achieving a very high oil content determined by Soxhlet extraction (34.93%).

Thus, we can conclude that the yield of cannabis seed extracts is influenced by various factors, including drying methods, which affect both mass loss and yield, the origin of the seeds, cultivation techniques, the planting to harvesting period, material handling, and agronomic and environmental conditions.

### 3.3 Phytochemical Study

**FIGURE 3:**
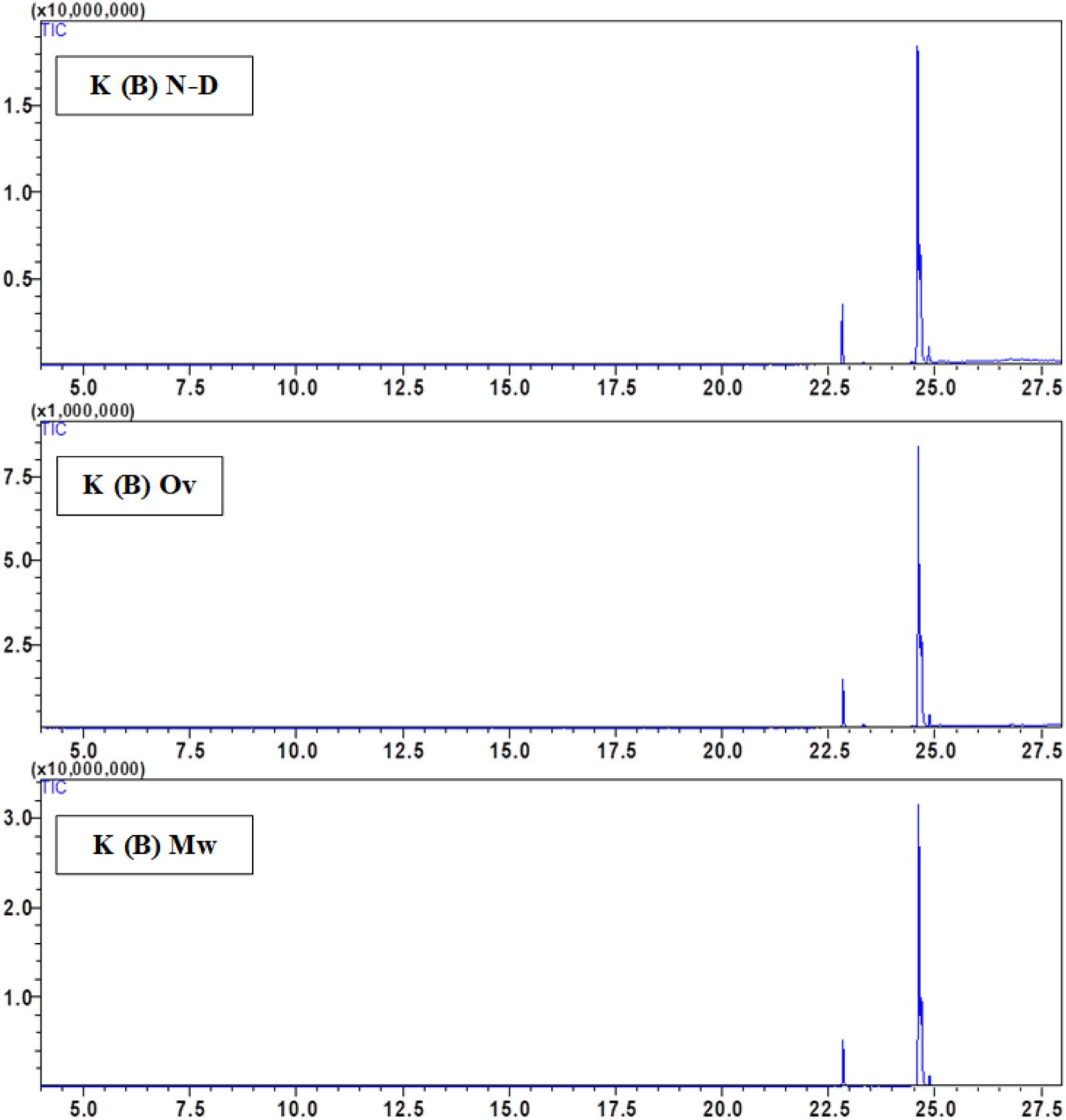
GC–MS chromatogram of the hexane extract of *Cannabis sativa* Beldia from Ketama (K(B)) using oven‐dried, microwave‐dried, and non‐dried seeds.

**FIGURE 4:**
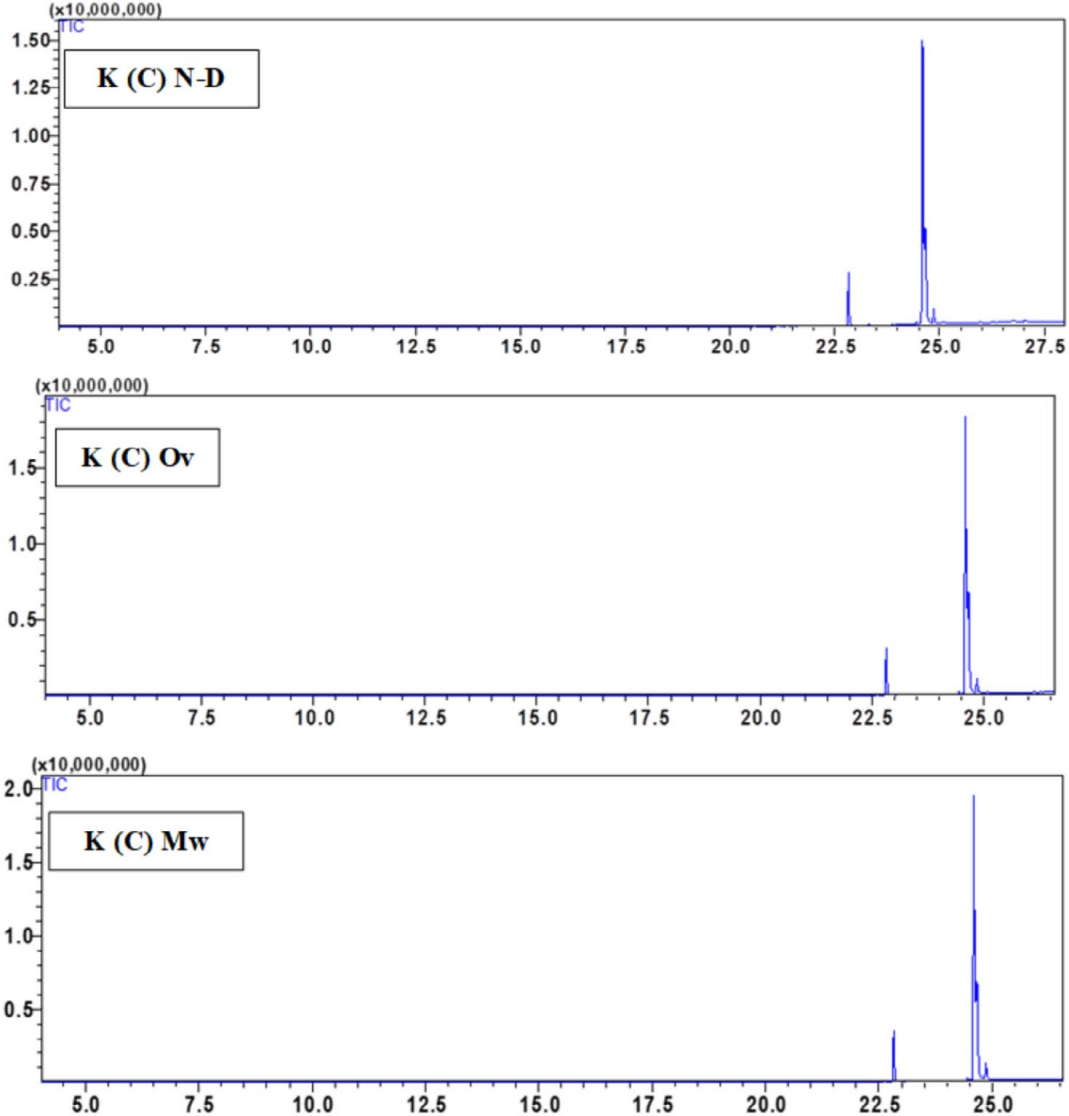
GC–MS chromatogram of the hexane extract of *Cannabis sativa* critical from Ketama K(C) using oven‐dried, microwave‐dried, and non‐dried seeds.

**FIGURE 5:**
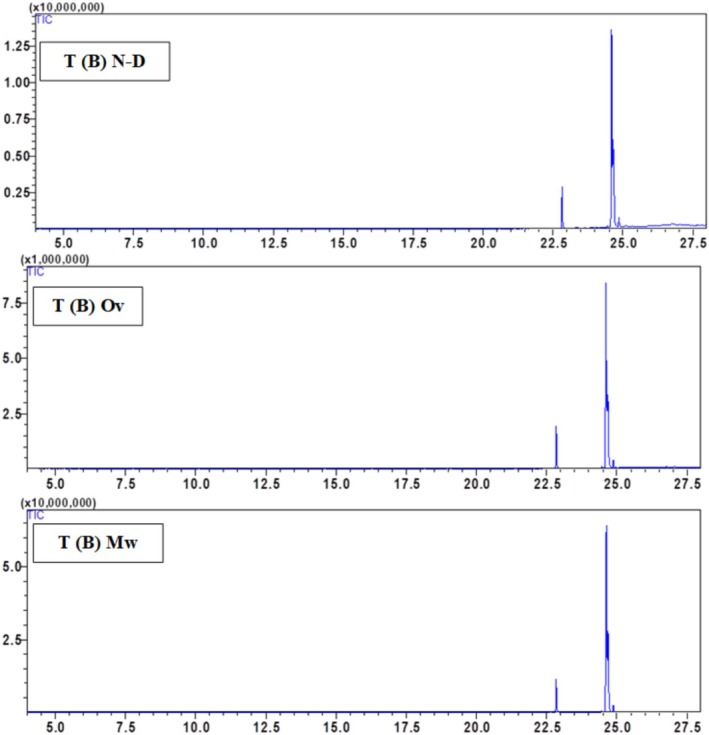
GC–MS chromatogram of the hexane extract of *Cannabis sativa* Beldia from Taounate T(B), using oven‐dried, microwave‐dried, and non‐dried seed.

**FIGURE 6:**
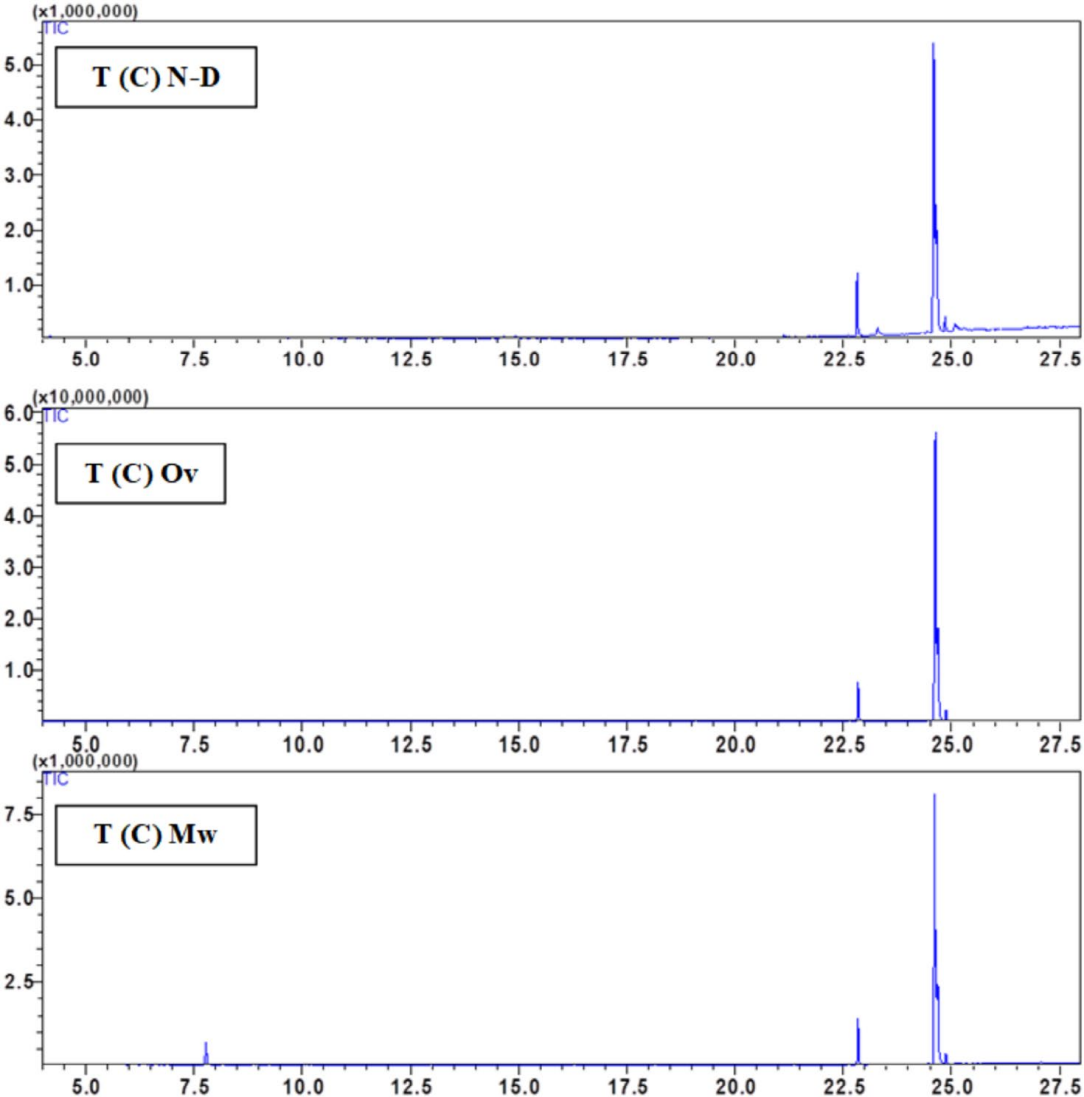
GC–MS chromatogram of the hexane extract of *Cannabis sativa* critical from Taounate T(C) using oven‐dried, microwave‐dried, and non‐dried seeds.

The chemical composition of the hexane seed extract of Beldia and Critical varieties from Ketama and Taounate shows significant variations depending on the drying method used (non‐dried (N‐D), oven‐dried (Ov), and microwave‐dried (Mw)) Table 2. The major compounds identified include palmitic acid, linolenic acid, linoleic acid, oleic acid, and stearic acid, with a minor presence of α‐pinene in the T(C) sample. Fatty acid composition varies depending on the drying method, with linoleic acid being the most abundant compound in all samples. In general, drying influences the relative abundance of fatty acids, likely due to thermal degradation or oxidation processes. Linoleic acid (C18:2) is dominant in all samples, particularly in K(C) (79.65%) and T(B) (73.13%) under non‐dried conditions. However, oven drying increases its proportion in some cases (e.g., K(B) from 50.88% to 66.59%). In contrast, microwave drying tends to decrease its relative content, as seen in K(C) (66.4%–79.27%) and T(C) (69.6%–50.63%), suggesting that microwave drying may induce partial degradation or isomerization. Oleic acid (C18:1) is particularly high in K(B) (35%) and T(B) (39.04%) under non‐dried conditions. Still, its proportion decreases with oven drying (e.g., from 35% to 20.95% in K(B) and from 39.04% to 38.99% in T(B)). In contrast, microwave drying shows variable effects, either increasing or decreasing the content depending on the sample, likely due to oxidation. The palmitic acid (C16:0) content changes across drying methods appear less drastic than in unsaturated fatty acids, remaining within a relatively stable range (7.17%–14.87%). However, an increase is observed in some cases, such as in T(B) with oven drying (11.23%) compared to non‐dried (9.29%), indicating a potential concentration effect due to water loss. Stearic acid (C18:0) shows a slight decrease in content with drying, for instance, in T(C), where it is reduced from 8.11% (N‐D) to 2.52% (Ov and Mw), possibly due to thermal degradation at high temperatures. α‐Pinene is only detected in the T(C) sample under microwave drying (5.12%), suggesting that microwave treatment may enhance the release of volatile compounds or facilitate their extraction due to the increased permeability of the seed matrix. However, its absence in other samples indicates that its formation might depend on the specific biochemical composition of the Taounate Critical variety. Regarding the overall impact of drying methods, oven drying generally increases linoleic acid content while decreasing oleic and stearic acid levels, indicating a selective impact on unsaturated fatty acids, possibly due to oxidation. Microwave drying tends to reduce linoleic acid content. It variably affects oleic acid, and detecting α‐pinene in T(C) suggests that microwave drying may induce chemical changes that release certain volatile compounds. Non‐dried samples tend to have higher oleic acid content and lower linoleic acid proportions than dried counterparts, highlighting that drying methods significantly alter lipid composition. The study emphasizes that drying methods profoundly impact the chemical composition of hexane seed extracts, with oven drying favoring polyunsaturated fatty acids like linoleic acid. In contrast, microwave drying can lead to volatile compound release. The variability in oleic and stearic acid content across treatments suggests that thermal processing influences lipid stability. Further studies on oxidative stability and bioactivity are recommended to optimize drying conditions to preserve the nutritional quality of these extracts (Figures 3, 4, 5, 6).

**TABLE 2:** Chemical composition of no dried (N‐D) and dried using oven (Ov) and microwave (Mw) in hexane seeds extract of Beldia from Ketama K(B), Critical from Ketama K(C), Beldia from Taounate T(B) and Critical from Taounate T(C) in percentage (%).

No.	Compounds	RT	K(B)			K(C)			T(B)			T(C)		
			N‐D	Ov	Mw	N‐D	Ov	Mw	N‐D	Ov	Mw	N‐D	Ov	Mw
1	Alpha, ‐Pinene	7.77												5.12
2	Palmitic acid	22.84	9.28	8.09	8.67	10.08	7.46	7.57	9.29	11.23	8.14	14.87	7.17	7.76
3	Linolenic acid	24.50	0.51	1.51	0.28	0.21	0.45	0.37	0.46		0.42	3.89	0.35	
4	Linoleic acid	24.61	50.88	66.59	55.56	79.65	66.4	79.27	47.63	47.26	52.92	73.13	69.6	50.63
5	Oleic acid	24.65	35	20.95	32.84	8.02	21.72	7.57	39.04	38.99	36.66		20.36	33.06
6	Stearic acid	24.87	4.33	2.86	2.65	2.02	3.73	4.69	2.98	2.52	1.86	8.11	2.52	2.43

Abbreviation: Nd, not detected.

**FIGURE 7:**
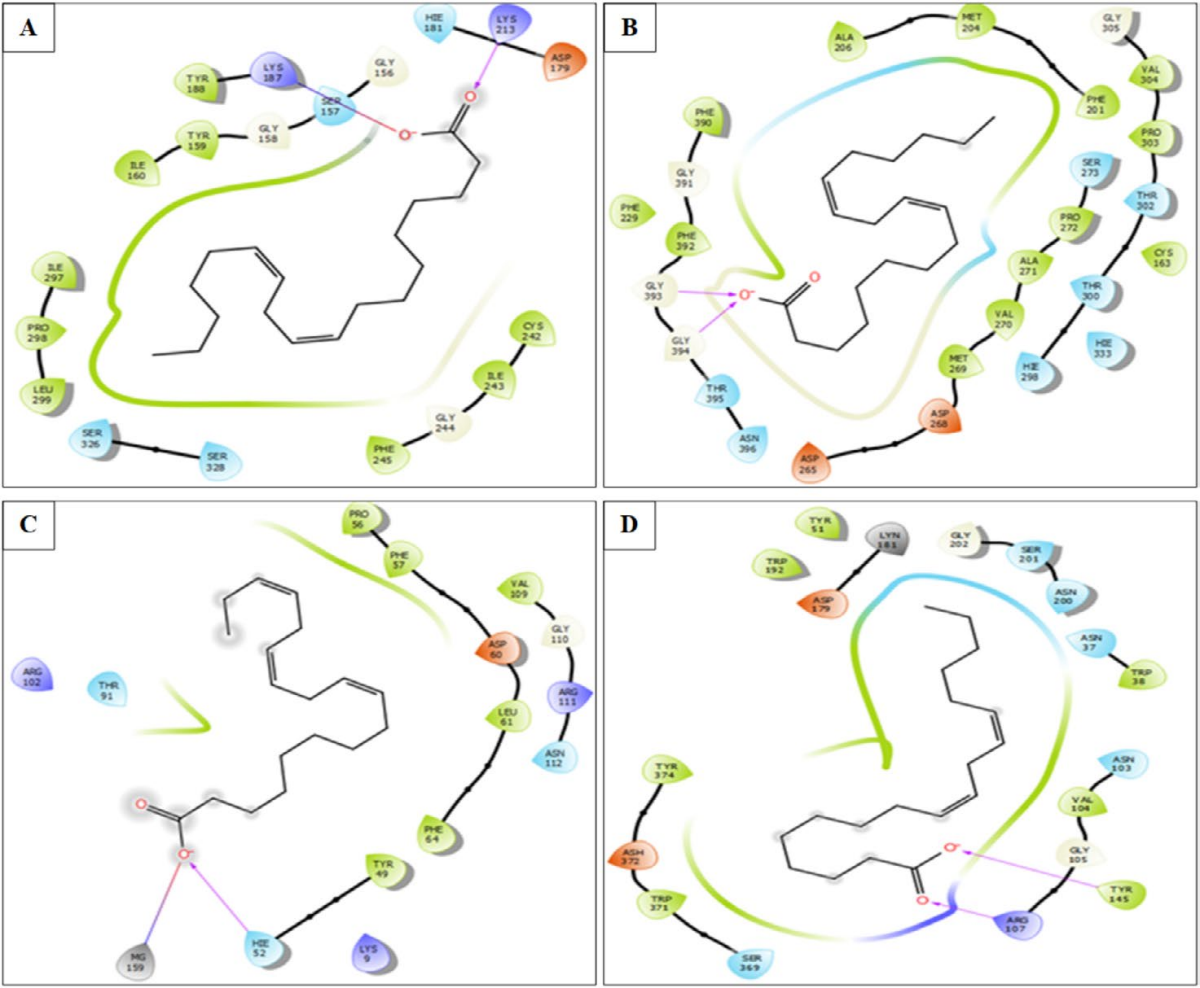
The 2D viewer of Linoleic acid interactions with the active site. (A): NADPH oxidase, (B): Beta‐ketoacyl‐[acyl carrier protein] synthase from *Escherichia coli* . (C): *Staphylococcus aureus* nucleoside diphosphate kinase. (D): *G. candidum* Cel7A structure.

**FIGURE 8:**
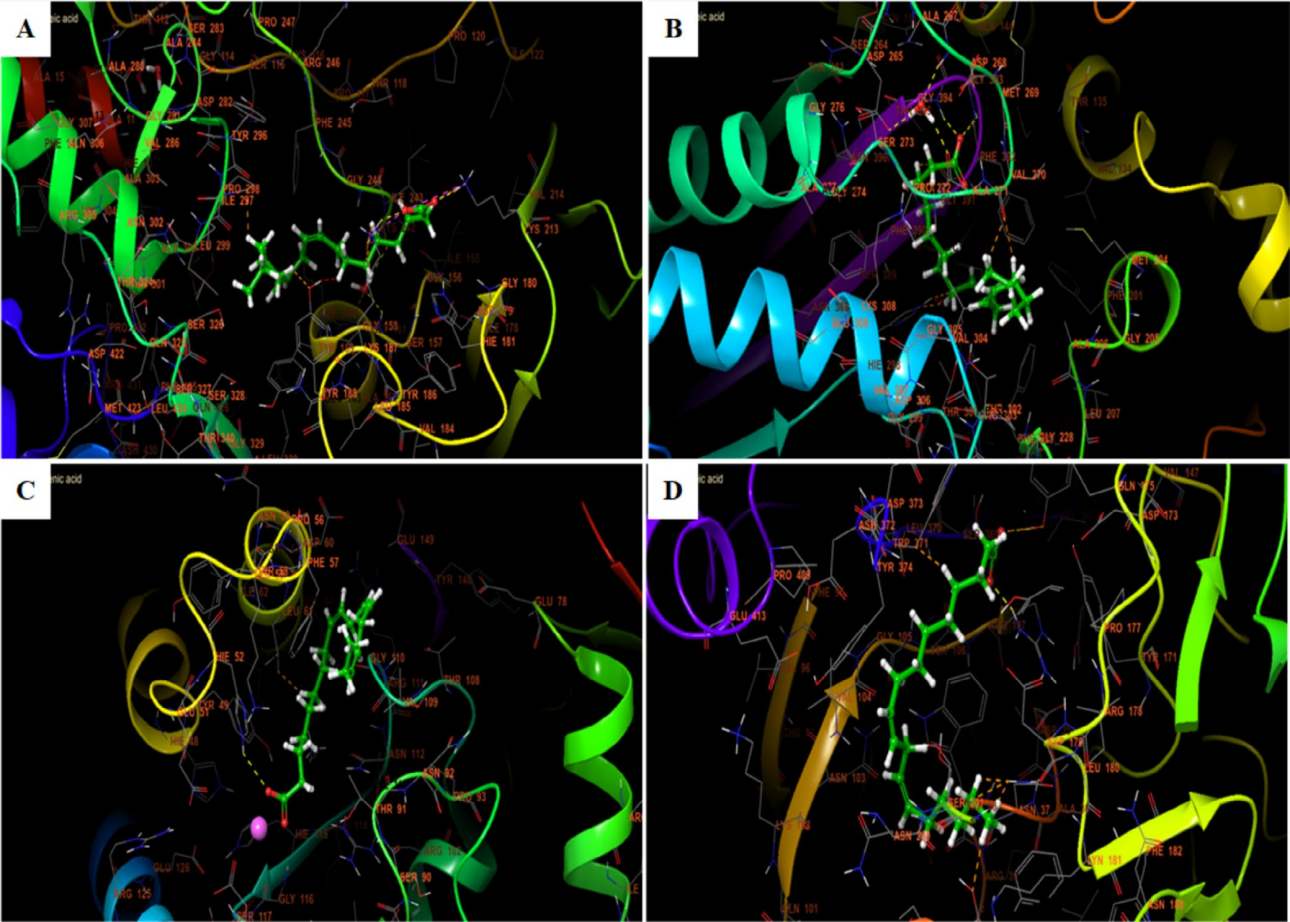
The 3D viewer of linoleic acid interactions with the active site. (A) NADPH oxidase, (B) beta‐ketoacyl‐[acyl carrier protein] synthase from *Escherichia coli* , (C) *Staphylococcus aureus* nucleoside diphosphate kinase, (D) *G. candidum* Cel7A structure.

**FIGURE 9:**
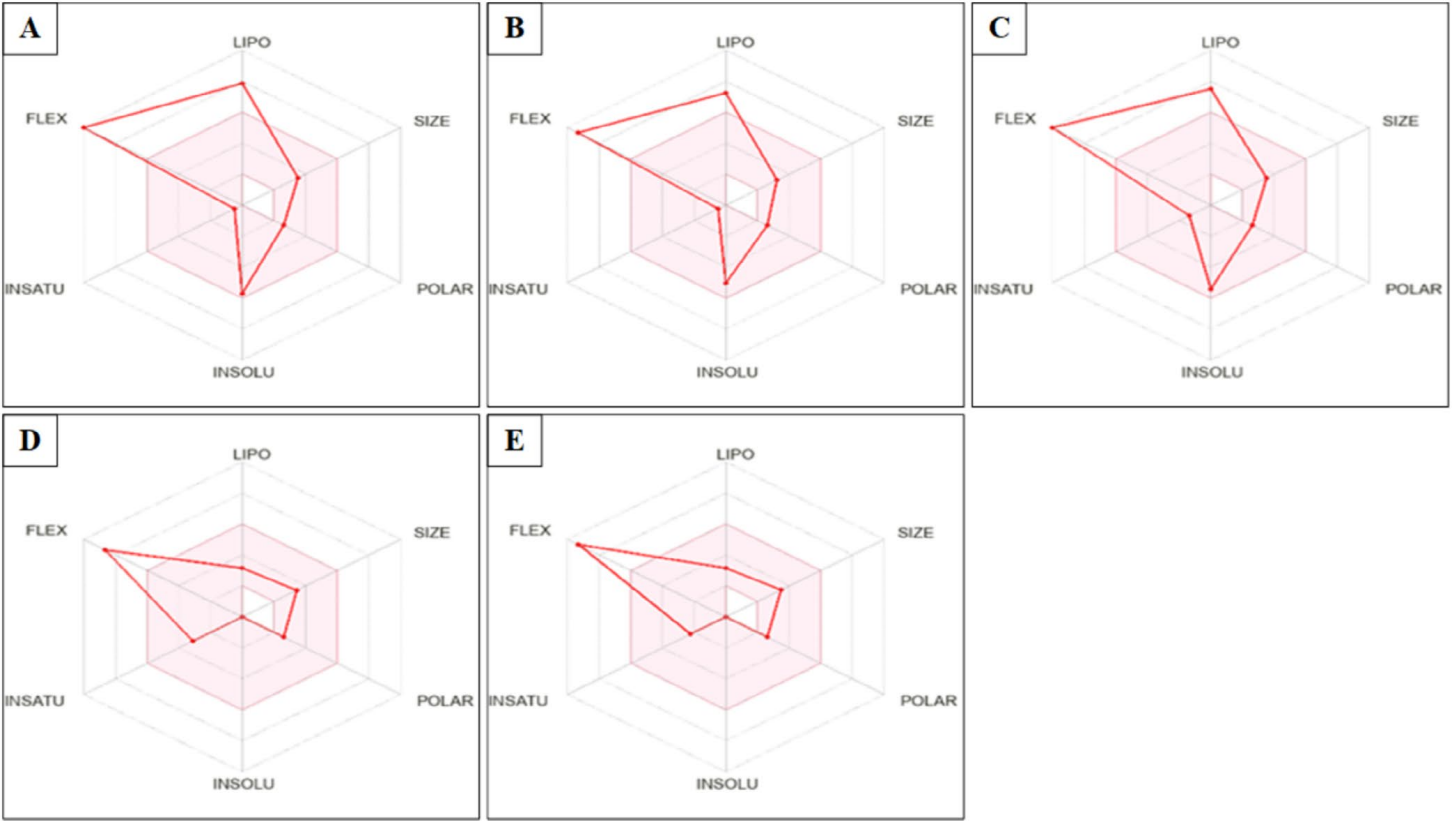
Bioavailability radars of the hexane extract of *Cannabis sativa* L. seed (A: Stearic acid; B: Palmitic acid; C: Oleic acid; D: Linolenic acid; E: Linoleic acid).

According to a study conducted by Haddou, Hassania Loukili, et al. (2023) on the characterization of *C. sativa* L. seed oil cultivated in northern Morocco, specifically from Ketama, it has been shown that cannabis seed oil contains several fatty acids, with linoleic acid as the primary compound, accounting for 42.92%, followed by 7‐octadecenoic acid (22.91%), palmitic acid (15.37%), linolenic acid (12.42%), stearic acid (4.74%), and finally, heptacosanoic acid at a percentage of 1.64%. Another study conducted by Orhan et al. (2000) on hemp seed oils showed relatively high levels of linoleic acid at 54.66%, followed by alpha‐linolenic acid with a percentage of 31.72%, palmitic acid at 8.53%, stearic acid at 3.06%, and gamma‐linolenic acid at 2.01%.

Among the main fatty acids in *C. sativa* L. seeds, Özdemir et al. (2021) report that linoleic acid is the major compound with a percentage of 55.03%, followed by α‐linolenic acid at 18.29%, oleic acid at 15.12%, palmitic acid at 6.32%, stearic acid at 2.87%, and γ‐linolenic acid at 0.54%. Additionally, Babiker et al. (2021) conducted a study on the chemical composition of fatty acids in *C. sativa* L. seeds, finding that linoleic acid constituted 58.52%, followed by α‐linolenic acid (16.44%), oleic acid at 16.12%, palmitic acid at 5.96%, and stearic acid at 2.56%.

### 3.4 Antioxidant Activities

Table 3 presents the IC_50_ values (in μg/mL) for the antioxidant activity of cannabis seed oils derived from two varieties, Beldia and Critical, collected from two regions, Ketama and Taounate. The results are reported for three drying conditions: non‐dried, oven‐dried, and microwave‐dried. The IC_50_ value of ascorbic acid, a standard reference antioxidant, is also provided.

**TABLE 3:** The antioxidant and free radical scavenging ability of hexane extracts.

Sample	DPPH scavenging capacity IC_50_ (μg/mL)				Total antioxidant capacity^a^			
	K(B)	K(C)	T(B)	T(C)	K(B)	K(C)	T(B)	T(C)
Non‐dried	9.61 ± 0.03	19.95 ± 0.02	85.97 ± 0.03	32.28 ± 0.02	451.39	529.37	391.64	470.03
Oven‐dried	31.185 ± 0.02	28.824 ± 0.05	80.614 ± 0.02	42.004 ± 0.03	442.69	435.60	387.25	398.60
Microwave‐dried	20.377 ± 0.04	18.486 ± 0.01	67.584 ± 0.03	55.883 ± 0.01	431.20	445.86	387.25	398.97
Ascotbic acid	61.43 ± 0.04				500.80			

^a^ Total antioxidant capacity expressed in μM ascorbic acid equivalents/mg oil.

The non‐dried seeds from the Ketama Beldia variety (K(B)) exhibit the lowest IC_50_ value (9.617 μg/mL), indicating the strongest antioxidant activity, followed by Ketama Critical (K(C)) with an IC_50_ value of 19.952 μg/mL. In contrast, the oils from non‐dried seeds from Taounate show significantly higher IC_50_ values, with Taounate Beldia (T(B)) at 85.979 μg/mL and Taounate Critical (T(C)) at 32.289 μg/mL, reflecting weaker antioxidant activity compared to Ketama seeds.

Drying methods impact the antioxidant potential of the seed oils. Oven drying significantly increases IC_50_ values, indicating reduced antioxidant activity. For K(B), the IC_50_ value rises to 31.185 μg/mL, whereas K(C) increases to 28.824 μg/mL. Similarly, the IC_50_ values for T(B) and T(C) remain high, with T(B) showing a particularly elevated value of 80.614 μg/mL.

Microwave drying shows a moderate effect, yielding IC_50_ values lower than those of oven‐dried samples but still higher than non‐dried ones. K(B) and K(C) retain relatively strong antioxidant activities, with IC_50_ values of 20.377 and 18.486 μg/mL, respectively. However, T(B) continues to exhibit a high IC_50_ value (67.584 μg/mL), whereas T(C) increases to 55.883 μg/mL.

The IC_50_ value for ascorbic acid is 61.34 μg/mL. This indicates that the non‐dried Ketama seed oils outperform ascorbic acid in terms of antioxidant activity, whereas dried samples, particularly those from Taounate, show diminished activity.

In summary, the cannabis seed oils from the Ketama region, especially the non‐dried Beldia variety, demonstrate superior antioxidant properties compared to those from Taounate. Drying, particularly oven drying, has a detrimental effect on the antioxidant potential of the oils, whereas microwave drying is less harmful.

According to a study conducted by Ahmed et al. (2019) on the total phenolic and flavonoid contents and the antioxidant activities of *Citrullus colocynthis* L. and *C. sativa* L., the best DPPH (%) inhibitions were recorded by aqueous and acetonic extracts, ranging from 34.20% ± 1.10% to 55.57% ± 1.20%. Another study by Pellegrini et al. (2021) showed that essential oils from Hemp had significant antioxidant potential when evaluated using the DPPH test. Additionally, a study by Yan et al. (2015) on the characterization of lignanamides from hemp seeds ( *C. sativa* L.) and their antioxidant and acetylcholinesterase inhibitory activities revealed a vigorous DPPH radical scavenging activity for cannabis seeds, with inhibitions ranging from 69.1% to 86.9% at a concentration of 100 μg/mL, comparable to the positive control quercetin.

Table 3 presents the concentrations of specific substances in hexane extracts of Beldia and Critical hemp seeds from both Ketama and Taounate regions, evaluated under three drying conditions: non‐dried, oven‐dried, and microwave‐dried.

The concentration of specific compounds in the non‐dried samples was highest, with K(C) exhibiting a notable 529.37 μg/mg level. In comparison, T(B) and T(C) showed lower concentrations of 391.64 and 470.03 μg/mg, respectively. This indicates that fresh seeds retain more bioactive compounds, essential for their antioxidant properties. Upon drying, both oven and microwave methods resulted in a reduction in concentrations across all samples. The oven‐dried K(B) and K(C) samples recorded concentrations of 442.69 and 435.60 μg/mg, indicating a slight decrease, whereas Ta(B) and Ta(C) samples showed even lower values at 387.25 and 398.60 μg/mg, respectively. This suggests that the drying process may lead to some loss of these beneficial compounds.

Interestingly, the microwave‐dried samples had comparable concentrations to oven‐dried samples, with Ke(B) at 431.20 and Ke(C) at 445.86 μg/mg. This indicates that microwave drying might be preferable as it appears to preserve the integrity of the compounds better than traditional oven drying.

The reference value for ascorbic acid, recorded at 500.805 μg/mg, exceeded all other samples, highlighting its potential as a benchmark for evaluating the antioxidant capacity of the cannabis extracts.

Overall, these findings underline the influence of drying methods on the retention of bioactive compounds in cannabis seeds, with non‐drying conditions showing the most beneficial effects on compound concentration.

Comparing our antioxidant test results with other studies on the TAC of *C. sativa* L. seed oils, several researchers have reported similar antioxidant potential. However, specific values vary based on extraction methods and plant parts analyzed. A study by Benkirane et al. found that optimized extraction of phenolic compounds from hemp seeds led to significant TAC levels, supporting the presence of hydroxycinnamic acid amides and lignan amides that contribute to the strong antioxidant properties in cannabis seeds. The TAC values achieved with an acetone‐water solvent mix were among the highest across several antioxidant tests (DPPH, ABTS, FRAP), underscoring cannabis seeds as a valuable source of natural antioxidants for potential health applications (Benkirane et al. 2022). Another study by Kubiliene et al. focused on the effects of cannabis extract on oxidative stress in vivo, reporting that cannabis oils decreased oxidative stress markers, such as malondialdehyde (MDA), whereas increasing glutathione (GSH) and catalase activity, which collectively support the antioxidant efficacy of *C. sativa* L. oils. This study aligns with our results showing a strong antioxidant profile, particularly in its protective effects against oxidative stress‐related damage (Kubiliene et al. 2021).

### 3.5 Antimicrobial Activities

Table 4 presents the antibacterial effects of different drying methods: non‐dried (ND), oven‐dried (OV), and microwave‐dried (Mw) on four varieties of *C. sativa* L.: Ketama Beldia (K(B)), Ketama Critical (K(C)), Taounate Beldia (T(B)), and Taounate Critical (T(C)). Each method's impact on antibacterial efficacy is observed through the inhibition zones against four bacterial strains: *S. aureus* , *M. luteus* , *E. coli* , and *P. aeruginosa* . Here, we compare the drying methods across varieties and strains.

**TABLE 4:** Antibacterial activity inhibition zones in (mm).

	*Staphylococcus aureus*	*Micrococcus luteus*	*Escherichia coli*	*Pseudomonas aeruginosa*
N‐D K(B)	12 ± 0.4	13 ± 0.1	11.5 ± 0.1	11 ± 0.3
OV K(B)	13 ± 0.2	13.2 ± 0.1	12 ± 0.1	12 ± 0.2
Mw K(B)	12 ± 0.2	13 ± 0.4	12.5 ± 0.4	11 ± 0.3
N‐D K(C)	13.1 ± 0.1	12 ± 0.2	13 ± 0.2	11.4 ± 0.1
OV K(C)	14 ± 0.3	13.7 ± 0.4	14 ± 0.2	12.8 ± 0.1
Mw K(C)	14.3 ± 0.1	13 ± 0.2	13.6 ± 0.1	14 ± 0.1
N‐D T(B)	13 ± 0.2	14 ± 0.1	12 ± 0.3	11 ± 0.1
OV T(B)	14 ± 0.1	15.3 ± 0.1	12.3 ± 0.1	12 ± 0.2
Mw T(B)	14.2 ± 0.5	15.1 ± 0.3	13 ± 0.4	12.5 ± 0.2
N‐D T(C)	13 ± 0.1	13.8 ± 0.1	11 ± 0.1	12.4 ± 0.2
OV TC)	13 ± 0.2	14.2 ± 0.1	13 ± 0.2	12.9 ± 0.2
Mw T(C)	13.5 ± 0.5	14.2 ± 0.2	13 ± 0.4	13 ± 0.2
T‐ DMSO	0 ± 0.0	0 ± 0.0	0 ± 0.0	0 ± 0.0
T+ Gentamicine	25 ± 1.2	21 ± 1.1	22 ± 0.9	25 ± 1.1

In the Ketama Beldia (K(B)) variety, there are notable differences between drying methods, particularly when comparing the ND, OV, and Mw samples. For *S. aureus* , all methods show similar antibacterial effects with inhibition zones ranging around12 to 133 mm, yet the OV treatment slightly outperforms ND and Mw by 1 mm, indicating a marginal enhancement. This trend is also reflected against *E. coli* and *P. aeruginosa* , with the inhibition zones increasing slightly from ND to OV and Mw. However, the changes are minimal, suggesting that the drying method does not drastically alter the antibacterial potency in K(B).

The Ketama Critical (K(C)) variety shows more prominent changes with the different drying methods. Here, OV and Mw drying display superior antibacterial activity compared to ND, particularly with *M. luteus* and *E. coli* , where the OV method achieves inhibition zones up to 1.7 mm larger than the ND method, and Mw displays similar enhancements. This indicates that drying through OV and Mw can significantly boost the antibacterial effects for K(C), which may suggest a potential modification in the active components due to heat exposure, especially notable in the increased inhibition for both *S. aureus* and *P. aeruginosa* .

The differences across drying methods are also distinct in the Taounate Beldia (T(B)) samples. The Mw drying method shows a consistently higher antibacterial activity, with inhibition zones for *M. luteus* reaching 15.1 mm, surpassing both ND and OV. Similarly, for *E. coli* , the Mw drying method again displays a slight increase, showcasing a potential enhancement effect through microwave drying for this strain. Although all methods exhibit moderate antibacterial activity, Mw‐treated samples generally achieve the highest inhibition, particularly against *S. aureus* and *P. aeruginosa* , indicating that the Taounate Beldia variety may respond more to microwave‐induced drying effects.

Lastly, for the Taounate Critical (T(C)) variety, the results show that the drying method influences antibacterial activity differently across bacterial strains. Both OV and Mw methods enhance inhibition against *M. luteus* compared to ND, achieving 14.2 and 13.5 mm, respectively. However, for *E. coli* , the results vary minimally across all drying methods, indicating limited impact on this strain. For *P. aeruginosa* , however, OV treatment seems most effective, followed closely by Mw. This suggests that while drying methods like OV can enhance antibacterial effects for T(C), their influence may be strain‐dependent.

In conclusion, these findings illustrate that drying methods affect antibacterial activity in *C. sativa* L., with variations among the varieties and strains. Generally, OV and Mw treatments tend to slightly enhance antibacterial effects compared to ND, likely due to the activation or preservation of antibacterial compounds that may be sensitive to moisture or heat alterations. The degree of enhancement varies across varieties and bacterial strains, with Mw often showing promising results, particularly for *M. luteus* and *P. aeruginosa* , potentially offering an efficient method for improving antibacterial properties in certain cannabis varieties.

A study by Juliano et al. analyzed hemp oils from two cultivars. They effectively inhibited Gram‐positive strains, particularly with components such as cannabidiol (CBD) and myrcene contributing to the antibacterial properties. This aligns with the high efficacy of oven‐ and microwave‐dried cannabis extracts in inhibiting *S. aureus* in the Moroccan varieties, where higher CBD concentrations could correlate with more substantial antibacterial results (Juliano et al. 2024). Comparatively, Ali et al. demonstrated that cannabis seed oil extracts exerted significant inhibition zones for *S. aureus* and *B. subtilis* , highlighting the effectiveness of hemp oils against Gram‐positive organisms due to bioactive compounds like terpenes. The study also showed moderate activity against *E. coli* , similar to what was observed with non‐dried (ND) Moroccan cannabis varieties, suggesting that non‐dried cannabis may contain lower levels of the specific antibacterial compounds enhanced through drying treatments (Ali et al. 2012).

Table 5 presents the antifungal activity of *C. sativa* L. across different drying methods (non‐dried (N‐D), oven‐dried (OV), and microwave‐dried (Mw)) and varieties (Ketama Beldia (K(B)), Ketama Critical (K(C)), Taounate Beldia (T(B)), and Taounate Critical (T(C))) shows notable variations in inhibition zones against *G. candidum*, *A. niger*, *C. glabrata*, and *R. glutinis* . Each drying method influences the antifungal efficacy differently, reflected in the observed inhibition zones.

**TABLE 5:** Antifungal activity inhibition zones in mm.

	*Geotrichum candidum*	*Aspergillus niger*	*Candida glabrata*	*Rhodotorula glutinis*
N‐D K(B)	13.2 ± 0.4	12.9 ± 0.1	13 ± 0.3	11.5 ± 0.1
OV K(B)	14.5 ± 0.2	14 ± 0.2	14 ± 0.2	12.6 ± 0.2
Mw K(B)	13.8 ± 02	13.5 ± 04	14.2 ± 02	12 ± 01
N‐D K(C)	14 ± 0.1	13.1 ± 0.1	12 ± 0.1	14.2 ± 0.2
OV K(C)	14.6 ± 0.3	15 ± 0.2	12.9 ± 0.3	15 ± 0.3
Mw K(C)	14 ± 0.3	14.1 ± 0.2	13.5 ± 0.4	15 ± 0.1
N‐D T(B)	14.9 ± 0.2	12.5 ± 0.1	13 ± 0.1	13 ± 0.3
OV T(B)	15.5 ± 0.2	13 ± 0.5	15 ± 0.2	14 ± 0.3
Mw T(B)	15.7 ± 0.1	14.5 ± 0.1	14 ± 0.2	14 ± 0.2
N‐D T(C)	13 ± 0.2	14 ± 0.1	13.1 ± 0.1	13.8 ± 0.1
OV TC)	14 ± 0.1	15.3 ± 0.1	14 ± 0.3	14.2 ± 0.1
Mw T(C)	14.2 ± 0.5	15.1 ± 0.3	14.3 ± 0.1	14.2 ± 0.2
Gentamicine	20 ± 1.2	22 ± 1.0	23 ± 0.5	24 ± 1.3

For the Ketama Beldia (K(B)) variety, non‐dried samples exhibit moderate antifungal activity across all fungi, with inhibition zones ranging from 11.5 to 13.2 mm. However, both oven‐dried and microwave‐dried methods increase the inhibition zones slightly. Oven drying enhances activity against *G. candidum* (14.5 mm) and *A. niger* (14 mm) more than microwave drying, although microwave drying shows a slight advantage against *C. glabrata* (14.2 mm). This suggests that oven drying in K(B) potentially enhances specific antifungal components, especially for fungi like *G. candidum* and *A. niger* .

In the Ketama Critical (K(C)) variety, non‐dried samples present moderate inhibition zones, such as 14 mm for *G. candidum* and 13.1 mm for *A. niger*. When K(C) is subjected to oven drying, there is an increase in inhibition against all tested fungi, reaching 15 mm against *A. niger* and *R. glutinis* . Microwave drying also shows strong activity, particularly against *R. glutinis* , with a zone of 15 mm. This trend indicates that both drying methods enhance the antifungal potency in K(C), especially for *A. niger* and *R. glutinis* .

For the Taounate Beldia (T(B)) variety, non‐dried samples show inhibition zones ranging from 12.5 to 14.9 mm, with *G. candidum* having the highest sensitivity. Oven drying significantly boosts activity across all strains, particularly against *G. candidum* (15.5 mm) and *C. glabrata* (15 mm). Microwave drying continues to show strong activity, with inhibition zones for *G. candidum* and *A. niger* at 15.7 and 14.5 mm, respectively. These results suggest that drying methods, especially microwave drying, enhance the antifungal potential of T(B) extracts, likely concentrating compounds with specific activity against *G. candidum*.

Taounate Critical (T(C)) also demonstrates varied antifungal responses. Non‐dried samples show inhibition zones ranging from 13 to 14 mm, with *C. glabrata* and *A. niger* exhibiting similar sensitivity. Oven drying improves these values, particularly against *A. niger* (15.3 mm) and *R. glutinis* (14.2 mm). Microwave drying also enhances antifungal efficacy, achieving a maximum inhibition zone of 15.1 mm for *A. niger* . The results indicate that, similar to other varieties, drying methods bolster the antifungal activity in T(C), with oven drying showing slightly higher efficacy for *A. niger*.

In summary, the oven and microwave drying processes generally enhance antifungal activity across all four *C. sativa* varieties. Oven drying often shows slightly better results for certain fungi, such as *A. niger*, whereas microwave drying may concentrate bioactive compounds to improve inhibition against fungi like *G. candidum*. This enhancement could be attributed to preserving and concentrating specific antifungal compounds, such as cannabinoids and terpenes, during drying.

The results of this study on the antifungal activity of *C. sativa* L. are consistent with previous findings that report moderate antifungal activity in extracts from cannabis seeds against various microbes. Specifically, studies have shown that cannabis seed extracts generally demonstrate a limited antifungal range. For instance, Esra et al. observed that cannabis seed oil exhibited no antifungal activity. At the same time, methanol extracts from the entire plant produced minor activity against *Candida albicans* (13 mm inhibition zone), with no activity against *A. niger* (Ali et al. 2012). Furthermore, research by Haddou et al. analyzed the biological activities of Moroccan cannabis seed extracts, revealing that ethanol extracts presented a relatively high inhibition zone (23 mm) against *Penicillium* sp. This suggests that, depending on the solvent used for extraction, certain cannabis compounds may vary significantly in their antifungal efficacy, potentially due to differences in their chemical profiles and bioactive compound concentrations. Ethanol, in particular, may effectively extract cannabinoids and terpenes that contribute to antifungal activity, as shown in Haddou's study (Haddou, Mounime, et al. 2023). These comparative studies underline the variability of *C. sativa* L. antifungal potency based on extraction methods and solvents, aligning with the present study's findings on enhanced activity through specific drying processes that likely concentrate or preserve key antifungal compounds in cannabis.

Table 6 shows the MIC and minimum bactericidal concentration (MBC) values for *C. sativa* L. varieties against *S. aureus* , *M. luteus* , *E. coli* , and *P. aeruginosa* .

**TABLE 6:** Minimum inhibitory concentration (MIC) and minimum bactericidal concentration (MBC) values in %.

	*Staphylococcus aureus*		*Micrococcus luteus*		*Escherichia coli*		*Pseudomonas aeruginosa*	
	MIC (%)	MBC (%)	MIC (%)	MBC (%)	MIC (%)	MBC (%)	MIC (%)	MBC (%)
N‐D K(B)	2	16	1	8	4	≥ 16	4	≥ 16
OV K(B)	1	8	1	8	2	16	2	16
Mw K(B)	2	16	1	8	2	16	4	≥ 16
N‐D K(C)	1	8	2	16	1	8	4	≥ 16
OV K(C)	0.5	4	1	8	0.5	4	2	16
Mw K(C)	0.5	4	1	8	1	8	0.5	4
N‐D T(B)	1	8	0.5	4	2	16	4	≥ 16
OV T(B)	0.5	4	0.25	2	2	16	2	16
Mw T(B)	0.5	4	0.25	2	1	8	2	16
N‐D T(C)	1	8	1	8	4	≥ 16	2	16
OV T(C)	1	8	0.5	4	1	8	2	16
Mw T(C)	1	8	0.5	4	1	8	1	8

The various drying methods for the Ketama Beldia (K(B)) clearly influence MIC and MBC values. Oven‐dried K(B) exhibits the lowest MIC values, with concentrations as low as 1% for *S. aureus* and *E. coli* . At the same time, the non‐dried version shows higher MIC values, particularly for *E. coli* and *P. aeruginosa* (4%). MBC values for oven‐dried K(B) also decreased notably, with 8% MBC for *S. aureus* compared to 16% for non‐dried and microwave‐dried versions. This suggests that oven drying enhances the antibacterial efficacy of K(B), possibly due to the concentration of bioactive compounds that act against bacterial growth.

The Ketama Critical (K(C)) variety demonstrates the most potent antibacterial activity across drying methods, with the lowest MIC values observed in oven‐dried and microwave‐dried samples. The oven‐dried K(R) has a particularly low MIC of 0.5% for *S. aureus* and *E. coli* , with MBC values also lower than the non‐dried version. Interestingly, microwave drying for K(C) also achieves similar effectiveness, with MIC values as low as 0.5% for *S. aureus* and MBC values of 4% for both *S. aureus* and *M. luteus* . These results suggest that both drying methods are effective for K(C), possibly enhancing or preserving compounds with strong bactericidal effects.

For the Taounate Beldia (T(B)) variety, oven drying again appears to be effective in reducing MIC values, with 0.25% for *M. luteus* and 2% for *E. coli* , whereas non‐dried samples require higher concentrations for inhibition and bactericidal effects (e.g., 4% MIC for *S. aureus* ). Microwave drying is comparable, though not as effective as oven drying for *M. luteus* , which maintains a 0.25% MIC. This pattern suggests that T(B) benefits from drying processes similarly, with an increase in antimicrobial potency, particularly for Gram‐positive bacteria.

Lastly, the Taounate Critical (T(C)) variety shows a pattern where both oven and microwave drying reduce MIC values, especially for *M. luteus* (0.5% in both oven and microwave drying). The MBC values for *E. coli* and *P. aeruginosa* remain relatively high across drying methods, particularly in the non‐dried samples. However, the decreased MIC values in oven‐ and microwave‐dried T(C) samples indicate that drying enhances the extract's ability to inhibit bacterial growth, particularly for Gram‐positive strains like *S. aureus* .

In summary, the drying methods, especially oven drying, generally reduce the MIC and MBC values across all cannabis varieties, particularly against Gram‐positive bacteria. This trend aligns with findings that drying concentrates on bioactive compounds, such as cannabinoids and terpenes, which can exert antibacterial effects. Gram‐negative bacteria, however, tend to show less sensitivity across samples, likely due to their outer membrane structure, which limits compound penetration.

The study of the antifungal efficacy of *C. sativa* L. seed extracts, particularly in terms of MIC and minimum fungicidal concentration (MFC), reveals significant variations based on the drying method employed, as shown in Table 7 below.

**TABLE 7:** Minimum inhibitory concentration (MIC) and minimum fungicidal concentration (MBC) values in %.

	*Geotrichum candidum*		*Aspergillus niger*		*Candida glabrata*		*Rhodotorula*. *Glutinis*	
	MIC (%)	MBC (%)	MIC (%)	MBC (%)	MIC (%)	MBC (%)	MIC (%)	MBC (%)
N‐D K(B)	1	8	2	16	1	8	4	≥ 16
OV K(B)	0.5	4	0.5	4	0.5	4	2	16
Mw K(B)	1	8	1	8	0.5	4	2	16
N‐D K(C)	0.5	4	1	8	2	16	0.5	4
OV K(C)	0.5	4	0.25	2	2	16	0.25	2
Mw K(C)	0.5	4	0.5	4	1	8	0.25	2
N‐D T(B)	0.5	4	2	16	1	8	1	8
OV T(B)	0.25	2	1	8	0.25	2	0.5	4
Mw T(B)	0.25	2	0.5	4	0.5	4	0.5	4
N‐D T(C)	1	8	0.5	4	1	8	1	8
OV T(C)	0.5	4	0.25	2	0.5	4	0.5	4
Mw T(C)	0.5	4	0.25	2	0.5	4	0.5	4

For the Ketama Beldia variety, the non‐dried extracts showed MIC values of 1% against *G. candidum* and 2% against *C. glabrata*, with 8% and 16% MFC values, respectively. When oven‐dried, the MIC values decreased to 0.5% for both *G. candidum* and *A. niger*, whereas MFC values were consistently lower at 4%. Microwave drying produced similar MIC values as the non‐dried extracts but showed improved antifungal action against *C. glabrata*, with an MFC value of 4%. These results suggest that the drying method significantly enhances the antifungal activity of Ketama Beldia, particularly with oven‐drying yielding the best results in terms of both MIC and MFC.

In contrast, the Ketama Critical variety exhibited slightly different responses. The non‐dried extracts showed MIC values of 0.5% for *G. candidum* and 1% for *C. glabrata*, with 4% and 16% MFC values. Similar to the Ketama Beldia, the oven‐dried extracts significantly lowered the MIC values to 0.5% across various fungal strains, and the MFC values dropped to 2%. Microwave drying maintained the low MIC values while further reducing MFC values, particularly for *C. glabrata* and *R. glutinis* . This suggests that both drying methods enhance the antifungal properties of Ketama Critical, with oven drying again proving more effective.

The Taounate Beldia and Taounate Critical varieties also demonstrated noteworthy results. For Taounate Beldia, the MIC values for non‐dried extracts were 0.5% for *G. candidum* and increased to 2% for *C. glabrata*, with corresponding MFC values of 4% and 16%. The oven‐dried extracts showed a significant decrease in MIC to 0.25% and MFC to 2%. Microwave drying produced the lowest MIC values of 0.25% for *G. candidum*, further establishing the effectiveness of drying methods in enhancing antifungal activity.

Overall, the findings indicate that the drying method has a pronounced effect on the antifungal efficacy of *C. sativa* seed extracts. Both oven and microwave drying methods generally enhanced the antifungal activity, as evidenced by lower MIC and MFC values across all four varieties. This highlights the importance of processing techniques in optimizing the therapeutic potential of *C. sativa* seed extracts against fungal pathogens.

Various studies on the MIC and MBC of *C. sativa* L. seed oils show moderate antibacterial effects, particularly against *S. aureus* , *M. luteus* , *E. coli* , and *P. aeruginosa* . For instance, findings by Chakraborty et al. (2018) report that the MIC of cannabis seed oil for *S. aureus* ranges from 1% to 2%, with MBC values reaching up to 16% for more resistant strains like *P. aeruginosa* . Studies by Haddou, Mounime, et al. (2023) on Moroccan cannabis oils also indicate similar MIC values, aligning with the present data on non‐dried, oven‐dried, and microwave‐dried cannabis variants showing comparable MIC and MBC values across these bacterial strains studies collectively highlight the moderate antimicrobial properties of cannabis seed oil, where drying and extraction methods might play a role in enhancing efficacy across different bacterial strains. The antifungal and antibacterial properties of *C. sativa* L. seed oil have been evaluated in multiple studies, mainly focusing on its MIC and MBC across various pathogens. Research indicates that the effectiveness of *C. sativa* extracts varies significantly by the part of the plant used and the pathogen targeted. A study on the hydroalcoholic extracts from *C. sativa* inflorescences revealed notable antimicrobial activity, especially against pathogens like *E. coli* and *Bacillus subtilis* , with specific sensitivity in dermatophyte strains, suggesting that these extracts could be optimized for therapeutic (Serventi et al. 2023). Similarly, another study highlighted the efficacy of different *Cannabis* extracts, such as methanolic extracts, in showing moderate activity against fungal strains like *Candida albicans* and *A. niger*, which aligns with your findings on MIC and MFC levels across drying treatments and varieties. The effectiveness varied based on the chemical profile, mainly due to cannabinoids like cannabidiol (CBD) and cannabidiolic acid, which have been shown to impact microbial viability (Hourfane et al. 2023). In summary, the research supports the concept that *C. sativa* L. extracts exhibit differential antimicrobial activity depending on the chemical composition and pathogen, underscoring its potential for antifungal and antibacterial applications. These results align with the variations in MIC and MFC values across drying methods, reflecting the critical role of extraction and processing methods in enhancing or diminishing antifungal efficacy in *C. sativa*. L. oils.

### 3.6 *In Silico* Prediction

In antioxidant activity, the compounds identified in the hexane extract of *C. sativa* L. Seed showed inhibitory activity expressed as glide score between −0.006 and −0.817 kcal/mol against NADPH oxidase. Although in antibacterial activity, Linoleic acid, Linolenic acid, and Oleic acid were the most active with a glide score of −2.322, −2.066, and −1.634 kcal/mol against beta‐ketoacyl‐[acyl carrier protein] synthase from *E. coli* . Furthermore, these molecules were the most active against *S. aureus* nucleoside diphosphate kinase with a glide score of −4.384, −4.543, and −3.107 kcal/mol. In antifungal activity, Linoleic acid, Linolenic acid, Oleic acid, Palmitic acid, and Stearic acid showed glide scores of −1.828, −1.517, −1.054, −0.130, and −0.895 kcal/mol, respectively, against *G. candidum* Cel7A (Table 8).

**TABLE 8:** Docking results of ligands in different receptors.

Title	Glide gscore (kcal/mol)			
	PDB ID: 2CDU	PDB ID: 1FJ4	PDB ID: 3Q8U	PDB ID: 4ZZT
Linoleic acid	−0.817	−2.322	−4.384	−1.828
Linolenic acid	−0.546	−2.066	−4.543	−1.517
Oleic acid	−0.665	−1.634	−3.107	−1.054
Palmitic acid	−0.006	−0.764	−2.649	−0.13
Stearic acid	−0.552	—	−2.88	−0.895

In the different active sites, linoleic acid establishes hydrogen bonds. In the active site of NADPH oxidase, a single hydrogen bond with residue LYS 213 and a salt bridge with residue LYS 187 was established. In the active site of beta‐ketoacyl‐[acyl carrier protein] synthase from *E. coli* , two bonds were established with residues GLY 393 and GLY 394. However, in the active site of *S. aureus* , nucleoside diphosphate kinase Linoleic acid established a single hydrogen bond with residue HIE 52 and a salt bridge with residue MG 159. In addition, it established in the active site of *G. candidum* Cel7A two hydrogen bonds with residues ARG 107 and TYR 145 (Figures 7 and 8).

### 3.7 ADME/Toxicity Analysis

The ADME/toxicity analysis of *C. sativa* L. seed hexane extract focused on the physicochemical properties, pharmacokinetics, and toxicity predictions of five key fatty acids: stearic acid, palmitic acid, oleic acid, linolenic acid, and linoleic acid.

Regarding the physicochemical properties, all the identified compounds have molecular weights within an acceptable range for drug‐like molecules, with values ranging from 256.42 to 284.48 g/mol. Additionally, the number of rotatable bonds ranges from 13 to 16, indicating flexibility in the molecular conformation. The hydrogen bond acceptors and donors remain constant for all compounds (2 and 1, respectively), influencing their solubility and permeability. Moreover, the lipophilicity (WLOGP) values range from 5.55 to 6.33, indicating that these compounds are highly lipophilic, which may significantly impact their absorption and distribution (Table 9).

**TABLE 9:** Physicochemical properties of the hexane extract of *Cannabis sativa* L. seed.

Formula	MW	Rotatable bonds	H‐bond acceptors	H‐bond donors	MR	TPSA	WLOGP
Stearic acid	284.48	16	2	1	90.41	37.3	6.33
Palmitic acid	256.42	14	2	1	80.8	37.3	5.55
Oleic acid	282.46	15	2	1	89.94	37.3	6.11
Linolenic acid	278.43	13	2	1	88.99	37.3	5.66
Linoleic acid	280.45	14	2	1	89.46	37.3	5.88

Figure 9 shows the bioavailability radars of *Cannabis sativa* L. seed hexane extract represented by the following parameters: lipophilicity (LIPO): −0.7 < XLOGP3 < +5, SIZE: 150 < MV < 500 g/mol, polarity (POLAR): 20 Å2 < TPSA < 130 Å2, insolubility (INSOLU): 6 < LOG S < 0, unsaturation (INSATU): 0.25 < Csp3 fraction < 1, and flexibility (FLEX): 0 < number of rotatable bonds < 9. The pink area is the appropriate physicochemical space for oral bioavailability, in which the graph of each molecule must be fully fitted to be declared as a drug.

All molecules investigated in this study, pnt showed flexibility values outside the pink area. Additionally, stearic acid, palmitic acid, and oleic acid showed flexibility and lipophilicity values outside the pink zone.

All compounds showed low water solubility regarding pharmacokinetic properties, which may limit their bioavailability. Furthermore, Caco‐2 permeability values suggest moderate absorption. Human intestinal absorption is > 90% for all compounds, suggesting good oral bioavailability. Notably, none of the compounds interact with P‐glycoprotein (P‐gp), reducing concerns about efflux‐mediated drug resistance (Table 10).

**TABLE 10:** Pharmacokinetics parameters of the hexane extract of *Cannabis sativa* L. seed.

			Stearic acid	Palmitic acid	Oleic acid	Linolenic acid	Linoleic acid
Absorption	Water solubility	Numeric (log mol/L)	−5.973	−5.562	−5.924	−5.787	−5.862
	Caco2 permeability	Numeric (log Papp in 10^−6^ cm/s)	1.556	1.558	1.563	1.577	1.57
	Intestinal absorption (human)	Numeric (% Absorbed)	91.317	92.004	91.823	92.836	92.329
	Skin permeability	Numeric (log Kp)	−2.726	−2.717	−2.725	−2.722	−2.723
	P‐glycoprotein substrate	Categorical (Yes/No)	No	No	No	No	No
	P‐glycoprotein I inhibitor	Categorical (Yes/No)	No	No	No	No	No
	P‐glycoprotein II inhibitor	Categorical (Yes/No)	No	No	No	No	No
Distribution	VDss (human)	Numeric (log L/kg)	−0.528	−0.543	−0.558	−0.617	−0.587
	Fraction unbound (human)	Numeric (Fu)	0.051	0.101	0.052	0.056	0.054
	BBB permeability	Numeric (log BB)	−0.195	−0.111	−0.168	−0.115	−0.142
	CNS permeability	Numeric (log PS)	−1.707	−1.816	−1.654	−1.547	−1.6
Metabolisme	CYP2D6 substrate	Categorical (Yes/No)	No	No	No	No	No
	CYP3A4 substrate	Categorical (Yes/No)	Yes	Yes	Yes	Yes	Yes
	CYP1A2 inhibitior	Categorical (Yes/No)	Yes	No	Yes	Yes	Yes
	CYP2C19 inhibitior	Categorical (Yes/No)	No	No	No	No	No
	CYP2C9 inhibitior	Categorical (Yes/No)	No	No	No	No	No
	CYP2D6 inhibitior	Categorical (Yes/No)	No	No	No	No	No
	CYP3A4 inhibitior	Categorical (Yes/No)	No	No	No	Yes	No
Excretion	Total clearance	Numeric (log mL/min/kg)	1.832	1.763	1.884	1.991	1.936
	Renal OCT2 substrate	Categorical (Yes/No)	No	No	No	No	No

In addition, the apparent volume of distribution (VDss) values are relatively low (negative values), suggesting limited tissue penetration. Moreover, the fraction unbound to plasma proteins is low for all compounds, indicating high plasma protein binding, which could reduce their free active concentrations. BBB permeability and central nervous system (CNS) permeability results show negative log values, further suggesting low penetration into the brain and central nervous system (Table 10).

Furthermore, in terms of metabolism, none of the fatty acids were identified as CYP2D6 substrates. Additionally, oleic acid, linolenic acid, and linoleic acid were predicted to be CYP3A4 substrates, indicating potential metabolism by this enzyme. Furthermore, linolenic acid was identified as a CYP3A4 inhibitor, which may lead to drug interactions if co‐administered with drugs metabolized by CYP3A4 (Table 10).

Clearance values range from 1.763 to 1.991 log mL/min/kg, suggesting moderate elimination. Moreover, none of the compounds interact with the renal transporter OCT2, reducing the risk of drug interactions related to renal clearance (Table 10).

Regarding toxicity predictions, none of the compounds showed mutagenic potential according to the Ames test. Additionally, the maximum tolerated dose values in humans were negative, indicating moderate safety. None of the fatty acids were predicted to inhibit hERG I or hERG II channels, reducing concerns about cardiotoxicity. Acute oral toxicity (LD_50_) values ranged from 1.406 to 1.441 mol/kg, suggesting relatively low acute toxicity. Similarly, chronic oral toxicity (LOAEL) values ranged from 3.115 to 3.33 log mg/kg_bw/day, indicating that long‐term exposure may have toxic effects at higher doses (Table 11).

**TABLE 11:** Toxicity predictions of the hexane extract of *Cannabis sativa* L. seed.

Toxicity									
AMES toxicity	Max. tolerated dose (human)	hERG I inhibitor	hERG II inhibitor	Oral rat acute toxicity (LD_50_)	Oral rat chronic toxicity (LOAEL)	Hepatotoxicity	Skin sensitisation	*T. pyriformis* toxicity	Minnow toxicity
Categorical (Yes/No)	Numeric (log mg/kg/day)	Categorical (Yes/No)	Categorical (Yes/No)	Numeric (mol/kg)	Numeric (log mg/kg_bw/day)	Categorical (Yes/No)	Categorical (Yes/No)	Numeric (log μg/L)	Numeric (log mM)
No	−0.791	No	No	1.406	3.33	No	Yes	0.65	−1.565
No	−0.708	No	No	1.44	3.181	No	Yes	0.84	−1.083
No	−0.81	No	No	1.417	3.259	No	Yes	0.676	−1.438
No	−0.84	No	No	1.441	3.115	Yes	Yes	0.722	−1.183
No	−0.827	No	No	1.429	3.187	Yes	Yes	0.701	−1.31

Furthermore, hepatotoxicity was predicted for linolenic and linoleic acids, suggesting a potential for liver toxicity. Additionally, skin sensitization was positive for all compounds except stearic and palmitic acids, indicating possible allergenic effects (Table 3).

Finally, environmental toxicity predictions revealed potential toxicity to *Tetrahymena pyriformis* and minnow, highlighting ecological concerns (Table 11).

### 3.8 Limitations of the Study

This study has several limitations. Firstly, although care was taken to ensure consistency in seed selection, natural variability in seed size, maturity, and moisture content may have influenced the results. Secondly, the drying methods were applied under specific conditions (microwave power, oven temperature, and durations), which may not fully represent other practical or industrial scenarios. Further research involving broader sampling and optimized drying parameters is warranted.

## 4 Conclusion

This study highlights the significant impact of drying methods on the chemical composition, yield, and biological activities of *C. sativa* L. seed oils. Microwave drying was associated with higher mass loss and improved oil yield for Taounate varieties, whereas oven drying was more beneficial for the Ketama varieties. The GC–MS analysis confirmed linoleic acid as the dominant fatty acid, with drying methods introducing variability in fatty acid profiles. Antioxidant activity was highest in non‐dried seeds, emphasizing the detrimental impact of drying, particularly oven drying, on antioxidant potential. Antimicrobial analyses highlighted the enhanced antibacterial and antifungal properties of oils from dried seeds, with oven and microwave drying proving effective for activating bioactive compounds. These findings underline the importance of processing techniques in optimizing the functional properties of *C. sativa* L. seed oils, suggesting promising applications in health, food, and pharmaceutical industries. Moreover, the efficiency and reduced energy demand of microwave drying position it as a green technology, offering sustainable perspectives for industrial processing.

## Author Contributions

Conceptualization, O.B. and E.H.L. and M.R.; methodology, O.B., E.H.L., M.C., and M.I.Y.; software, O.B., M.C., and M.I.Y.; validation, M.R., E.H.L., M.B., N.A., and A.E.; formal analysis, O.B. and M.I.Y.; investigation, M.R.; resources, M.R. and A.A.; data curation, O.B., M.I.Y., M.A., F.A.N., and A.A.Q.; writing – original draft preparation, O.B., M.I.Y., A.E., and E.H.L.; writing review and editing, A.E., N.A., M.R., E.H.L., M.I.Y., M.B., A.A., M.A., F.A.N., M.C., and A.A.Q.; visualization, A.E. and M.B.; supervision, M.R.; funding acquisition, M.A., F.A.N., and A.A.Q.; all authors have read and agreed to the published version of the manuscript.

## Conflicts of Interest

The authors declare no conflicts of interest.

## Data Availability

The original contributions presented in the study are included in the article. Further inquiries can be directed to the corresponding author.
